# Stifling stateness: The Assad regime’s campaign against rebel governance

**DOI:** 10.1177/0967010618768622

**Published:** 2018-04-11

**Authors:** José Ciro Martínez, Brent Eng

**Affiliations:** University of Cambridge, UK; University of California Berkeley, USA

**Keywords:** Civil war, counter insurgency, rebel governance, state theory, Syria, welfare

## Abstract

This article assesses the impact of the Assad regime’s aerial bombardment campaign on a frequently neglected component of Syria’s ongoing civil war: rebel governance. While analysis of the military and humanitarian ramifications of such attacks has been extensive, these perspectives fail to consider how the Assad regime’s counter insurgency efforts subvert governance practices by Syria’s diverse rebel groups. Drawing on performative approaches to the ‘state’, we argue that opposition groups’ daily enactments of ‘stateness’ via two key welfare services – bread and healthcare provision – constitute a historically inflected and locally grounded critique of the incumbent. When executed successfully, such enactments can stabilize relations between rulers and ruled while offering a vision of an alternative polity. They can also attract the attention of rivals. The Assad regime’s aerial bombing campaign of rebel-held areas is thus neither a haphazard military strategy nor simply the product of long-standing sectarian hatreds, but a deliberate tactic through which it seeks to destroy a key threat to its authority.

## Introduction

On the evening of 30 May 2016, fighter jets loyal to Bashar al-Assad carried out overnight airstrikes against the city of Idlib, a de facto rebel stronghold (Al Jazeera, 2016).^1^ The bombing targeted the public hospital and its surroundings, killing as many as 60 people and injuring over 100, according to local activists. Most of the victims were patients, women and children. Shortly afterwards, the director of the hospital stated that debris and wreckage covering the facility had put its operations out of service and forced its remaining staff underground (Shaheen, 2016). It was the fourth medical facility targeted by the Assad regime in two days. On 12 June, less than two weeks later, Syrian government warplanes struck a popular vegetable market in the same city. More than 30 civilians died and 50 were injured (Associated Press, 2016; The New Arab, 2016). These attacks targeted neither strategic military positions nor rebel fighters, yet they are indicative of a broader pattern witnessed throughout Syria’s civil war. Since the beginning of peaceful protests calling for the downfall of President Assad in April 2011, pro-regime forces have sought to suppress attempts by opposition groups to govern. The destruction of material infrastructure and the built environment has been the primary means through which this tactic has been pursued. In the countryside, forces loyal to the Assad regime burn crops, demolish wheat silos and destroy roads. In towns, combat aircraft target water tanks, market places and bakeries. Seemingly indiscriminate or unnecessarily cruel, this strategy has been both deliberate and openly proclaimed by regime-funded militias (*shabeeha*): ‘Either Assad or We’ll Burn the Country’ (*Assad aw nahraq al balad*) (Yassin-Kassab and Al-Shami, 2016: 106).

The Syrian government’s targeting of public infrastructure in opposition-held areas has been written about at great length. Analysts and international observers claim that attacking population centres has been a key pillar of the Assad regime’s military strategy (Hof, 2016; Hokayem, 2016; Fisher, 2016), while some scholars suggest that the destruction of infrastructure has emerged as a central aim in the war (Sowers et al., 2017: 416–419). The mainstream media has extensively covered the most brutal manifestations of this tactic, often focusing on human-interest stories and the cruel impact of aerial barrages on local residents (Nott, 2016; Rubin and Saad, 2016). While these and other accounts are undoubtedly important, we suggest that they often overlook the political logic underpinning the Syrian government’s systematic attacks. To understand this logic, we explore the bombing campaign’s repercussions from a different angle: rebel governance.^2^ We argue that concerns with the latter best explain the Assad regime’s targeted bombardment of rebel-held areas.

Building upon the work of several post-structural state theorists (Mitchell, 1991; Painter, 2006; Jeffrey, 2013; McConnell, 2016), our approach departs from the widely held conception of the state as a concrete institutionalization of power with measurable faculties. In our empirical analysis of state authorities and their rebel counterparts, we eschew the predominantly positivist approaches that dominate the study of civil war. By ascribing to the state an institutional identity and an assumed coherence, these frameworks not only produce a false distinction between state and society; they also obscure the everyday processes through which political authority is performed and experienced. Valuable as such analyses may be at pinpointing approximate causal relationships, their reliance on quantifiable forms of coercion or remuneration, exercised by a cohesive institutional body, means they cannot evaluate the micro-practices, symbolic acts and technologies of government that make a political authority both tangible and thinkable amongst civilians.

To avoid these limitations, we conceptualize the state and rebel governments as contingent political constructs; not concrete bodies with capacities to be measured but ‘the metaphysical effect of practices that make such structures appear to exist’ (Mitchell, 1991: 94). We theorize political authority, whether enshrined in the state or a rebel government, as an assemblage produced through everyday acts intended to convince an audience of particular sovereign arrangements. Consequently, this work is less interested in the state as a legal concept, independent variable or institution with capacities that determine civil war onset or outcomes (Fearon, 2004; Braithwaite, 2010; Sobek, 2010). We find state institutions and their capacities to be far too protean and elusive to take at face value, especially in a context such as civil war where multiple authorities compete to proclaim themselves as sovereign. Instead, we are interested in how opposition forces and incumbent authorities seek to shape political allegiances and consolidate their authority through everyday practices.

During the Syrian conflict, the enactment of state-like functions – what we term, drawing on the work of Alex Jeffrey (2013), ‘performing the state’ – has been one of the most important considerations for both rebel groups and the incumbent authorities that seek their defeat. These ‘state performances’ are articulated through institutions, signifiers and services that materially constitute and discursively (re)produce political authority. Examples from the Syrian war include establishing checkpoints that control the movement of people and goods, taxing local businesses, founding courts to resolve local disputes, coordinating agricultural production and organizing schooling. Such performances play a crucial role for rebel groups enmeshed in contexts of contested sovereignty. When executed successfully, they foster legitimacy and demonstrate an ability to govern proficiently while making explicit the contingent nature of the incumbent’s rule (Martínez and Eng, 2017). Importantly, certain performances can carry more symbolic value because of their association with historically specific manifestations of authority in a given place. Conversely, state forces have sought to disrupt these performances. By systematically annihilating the administrative institutions and public services that shape rebel-civilian relations, the Assad regime delegitimizes its competitors and prevents the emergence of coherent alternatives.^3^ Targeted aerial bombardment is especially effective in this respect. It works not only through inflicting military, financial and psychological damage, but also by interrupting and undermining everyday practices through which rebels generate local support and consolidate their rule.

This article will demonstrate these points in five parts. It begins by offering an overview of the literature on state military action and rebel governance during civil war before putting forth an alternative framework through which to analyse these phenomena. We then present a brief history of two key welfare services in Syria – healthcare and bread provision – so as to contextualize their salience during the current conflict. This section illustrates how pre-conflict interactions with state institutions shape the ways in which rebel groups have engaged in local governance. The third section introduces the rebel-held province of Idlib, which will be our focus. Sections four and five offer case studies of the Assad regime’s systematic targeting of the welfare services mentioned above. These two services offer different but complementary lenses through which to examine how pre-conflict legacies and wartime contextual factors, such as persistent bombardment or lack of resources, impact rebel groups’ abilities to provide welfare and, by extension, ‘perform the state’. We conclude by discussing how the Assad regime’s subversion of rebel governance has shaped the trajectory of the Syrian conflict before expanding on the broader insights this article may offer scholars of civil war.

The full scope of local governance in Syria is undoubtedly difficult to assess: the regime and its opponents regularly disseminate information, but the accuracy of such material is difficult to confirm. Drawing on scholarly and personal knowledge of the country, we attempt to assess wartime governance without direct access to Syria since 2011, due to security restrictions. Our research was conducted between 2013 and 2016. It draws on 83 in-depth interviews conducted by email, video conferencing, instant messaging and telephone with civilians, activists, Local Coordination Committee (LCC) members and journalists in ten of the country’s 14 governorates. Many were interviewed on more than one occasion. We additionally benefit from a robust qualitative sample of respondents collected outside of Syria, which includes 145 interviews with Syrian refugees recently arrived in Jordan or Lebanon, many of whom remain in contact with relatives in the country and some of whom travel back to Syria regularly. We supplement interview data with secondary source literature and open-source material ranging from NGO and international organizations’ reports to newspapers and YouTube videos. To bolster our claims, we reinforce our findings with those of reputable news sources. Almost all our informants inside Syria asked to remain anonymous in view of the personal and professional risks involved in providing information on the sensitive topics we discussed. Due to these constraints, we use pseudonyms and anonymous quotes for information collected in confidential interviews. Our assertions should all be considered in light of the serious limitations on qualitative research inside northern Syria. Undoubtedly several gaps remain and further research is needed.

## Beyond the reliance on force: Governance during civil war

While reliance on force may be an essential precondition for governance (Arjona et al., 2015: 3), it is neither a sustainable long-term strategy nor a reliable way of generating allegiance amongst local populations. As a result, rebel groups that achieve territorial control frequently form governing bodies to pacify, control and garner support amongst civilians.^4^ Such efforts generate new configurations of political order that are performed and stabilized through the everyday routines they engender. Over the past five years, a great deal of scholarship has explored how such configurations surface, develop and operate. Scholars have examined how rebels establish dispute-resolving mechanisms (Arjona, 2014), provide public goods (Stewart, 2017; Weinstein, 2006) and champion symbolic claims to statehood (Mampilly, 2015). Others have dissected the multi-scalar nature of insurgent administrations, which are always already imbricated in an array of transnational networks (Checkel, 2013; Linebarger, 2016). A key insight of this burgeoning rebel governance school is that the routinization of a comprehensive system of governance – including the development of civil institutions, informal and formal norms of interaction, and rules of behaviour – is crucial to the normalization of insurgent rule amongst civilians (Mampilly, 2015: 77).

Moreover, the establishment of administrative institutions and provision of public services by rebels has proven crucial to ‘the nature and scope’ of their challenges to state power (Mampilly, 2011: 55). Why? When armed actors regulate different aspects of civilian life successfully, they gain access to economic resources, civilian cooperation and recruits. In addition, rebel groups achieve an aura of legitimacy by foregrounding their authority within the routines and patterns of everyday life. They do so not simply through material inducements but also by way of symbolic efforts that give social meaning to their actions, promoting a shared frame of reference vis-à-vis civilian populations (Mampilly, 2015: 83; Selbin, 2010: 24). Effective governance aids in both material and symbolic endeavours; it is especially important for rebel groups as, unlike an established state authority, they cannot take their affiliations with a specific population for granted (Mampilly, 2015: 85; Wickham-Crowley, 1987: 493). Yet the shifting nature of military and practical constraints during conflict complicates rebels’ ability and willingness to enact such practices. Often, they must navigate between a desire to build more permanent governance structures – to ‘perform the state’ – and the flexibility needed to face shifts on and off the battlefield.

Incumbent governments also confront various choices when faced with insurgents. In past conflicts, state authorities have frequently engaged with civilians living under insurgent control through violence (Downes, 2007; Weinstein, 2006; Wood, 2008). By fostering insecurity, military actions can undermine the relationship between rebels and civilians (Wickham-Crowley, 2015). Unable to protect those they claim to rule, rebels are hard-pressed to find a receptive audience for their political projects. Yet terror and the arbitrary use of force run their own risks. In addition to tarnishing a government’s reputation in the international community, which can result in sanctions or outside intervention (Coggins, 2015: 210), indiscriminate violence can alienate local populations and further mobilize support for rebel groups (Kalyvas, 2006: 72 and 146). The other crucial component of counterinsurgency lies in winning the so-called ‘hearts and minds’ of non-combatants. A near-consensus exists regarding the means through which this is achieved: a combination of service provision, remunerative inducements, restrained use of force and information campaigns (Trinquier, 2006; Kilcullen, 2010). These strategies all point to one important consideration: the quiet centrality of non-combatants to wartime calculations (Lyall, 2009; Mampilly, 2011). Effective counterinsurgency thus depends on a deft touch: it hinges on adept military operations that weaken rebel groups and nonviolent actions that sway civilians. Yet still, a broader assessment of the literature suggests too strict a delineation between coercive, remunerative and symbolic measures. Targeted aerial bombardment, for example, does not solely work to weaken rebel brigades or improve prospects on the battlefield; it can also undermine the daily governance practices through which local allegiance is achieved. It is this process that the Assad regime’s bombing campaign seeks to disrupt.

Throughout the Syrian civil war, rebel attempts to ‘perform the state’ have been frequent, deliberate and purposeful. One of the most important of these everyday practices has been the provision of welfare, defined as the ‘direct or indirect facilitation of services and programs that promote well-being and security’ (Cammett, 2014: 12). We have chosen to focus on welfare because of its quotidian nature, material impact and symbolic significance. Welfare in Syria has an especially strong association with the state because of the Ba’ath regime’s interventionist legacy. In addition to aiding the livelihood of local residents, service provision works to build community by signalling membership in a polity (Cammett, 2014: 8). Furthermore, when rebels delineate specific domains of governance they create realms of activity that can be acted upon, thereby fostering imaginaries of a political authority in command of a distinct administrative sphere (Davis, 2009; McConnell, 2016: 13). This not only projects their power but proves them trustworthy, familiar and deserving of support (Wilson and McConnell, 2015). Everyday exchanges in healthcare, education, sanitation and subsistence goods are thus never simple material inducements; they also work to produce and reify political authority through citizens’ participation, both as actors and audience.

During the Syrian conflict, the tenuous and unstable nature of rebel governance means these practices have been more akin to what McConnell (2016) describes as ‘rehearsals of statehood’ – instances in which groups lacking de jure sovereignty over territory and people seek to prove their feasibility as rulers by governing effectively.^5^ As a heuristic, the concept of ‘rehearsal’ forces us to ponder both why statehood endures as an aspiration amongst rebel groups and how everyday practices work to further their objectives (McConnell, 2016: 165–166). Importantly, this goal does not have to be explicitly articulated in order to be effective; the very performance of certain practices associated with the state in a specific historical–cultural context constitutes a claim, one that anticipates and instates altered contexts for their future reception (Butler, 1997: 160). While Salwa Ismail (2011) has noted that protests were crucial to reimagining Syrian nationhood during the initial phase of the uprising, welfare provision has been a key medium through which the aspiration for an alternative political order has been expressed since the onset of civil war. In addition, welfare provision works to denaturalize the permanence of the Syrian state – a political authority striving to maintain its own coherence – as currently constituted, and thus functions as a powerful ‘form of critique and resistance’ (McConnell, 2016: 185) for opposition actors. Crucially, however, these critiques are intimately shaped by pre-conflict interactions between Syrians and the very state authorities rebel groups seek to overthrow. Despite the tendency to treat insurgencies as ahistorical phenomena, rebel rule is ‘always embedded in historically contingent values, norms, beliefs and forms of governance’ (Hoffman, 2015: 158).^6^ These patterns are engrained both amongst rulers and ruled. As a result, rebel groups frequently mimic the functions, routines and symbols of the state authorities they seek to replace; they are endogenous to the political orders against which they rebel, even as they aspire to overturn the prevailing socio-political order (Förster, 2010). The vast majority of our interviewees echoed these findings. They overwhelmingly relayed a certain set of expectations regarding the provision of basic welfare services by governing authorities. In more than 100 interviews, the two services most frequently mentioned were closely associated with the state in pre-war Syria: the provision of healthcare and subsidized bread.^7^

## Welfare services in Ba’athist Syria

Similar to several of its regional counterparts, the Assad-led Ba’athist regime (1970–) provided various forms of non-targeted social welfare to the citizenry, including free healthcare, education and subsidized food and utilities. These measures were part of a tacit social pact described by Sadiki as ‘*dimuqratiyyat al-khubz*’ (democracy of bread), in which public goods were ostensibly exchanged for political compliance (Sadiki, 1997: 135). Prior to the current conflict, healthcare in Syria was comprised of a government-run public system that provided the majority of primary care. Beginning in the late 1970s, the Health Ministry pursued broad-based vaccination coverage and dramatic increases in primary care access by establishing an extensive number of medical facilities throughout the country. These provided treatment for a wide range of medical conditions (Damascus Ministry of Health and WHO, 2011). A comprehensive social health insurance programme ensured the facilities distributed many medicines free of charge – a task made easier by the fact that the country produced nearly 90% of its pharmaceutical needs. Health indicators improved dramatically between 1970 and 2000, and slightly less so over the following decade.^8^ Although the 2001–10 period saw attempts to liberalize the healthcare system – measures that resulted in widening inequality of access to quality facilities – core services remained in the hands of the Ministry of Health.^9^ Steadily declining maternal mortality rates during 40 years of Assad-led Ba’athist rule point to Syria’s pre-conflict success in the healthcare sector compared to its regional counterparts, despite economic pressures and diplomatic isolation (Sen et al., 2013).

These successes have declined precipitously since 2011. The pharmaceutical industry, located amidst what quickly became contested areas (rural Damascus, Aleppo, Homs), is now defunct. This contributes to recurring shortages of vaccines, insulin, antibiotics and basic emergency supplies (Pitts-Tucker, 2012; Taleb et al., 2015). Even stable government-held areas are almost entirely dependent on medical supplies from foreign allies and UN aid. In rebel-held territories, especially those besieged by Assad-allied forces, the situation is often dire. By early 2013, small aid agencies and underfunded NGOs reliant on cross-border deliveries from Turkey and Jordan had become the main source of medicine and equipment. Beyond drug shortages, rebel-held areas are also deprived of health facilities and human resources by aerial attacks. According to the World Health Organization (WHO), 57% of Syria’s public hospitals have been damaged, while another 37% have been destroyed or closed. More than half of the country’s physicians have fled the country (Physicians for Human Rights, 2015). In formerly rebel-held parts of Aleppo, more than 95% of doctors have left. Life expectancy has dropped from 70 years in 2010 to 55 in 2015; 70,000 of the estimated 240,000 deaths during the conflict have resulted from a lack of medicines and adequate health services, especially hard-hitting for patients with chronic diseases (Karasapan, 2016). None of this has been accidental or inadvertent: ‘[The regime] bombs our hospitals to make life impossible’, said one Syrian activist who had recently fled to Beirut. ‘Many people are forced to leave the country because even a cold or the flu can become life-threatening’ (Interview 3).

The importance of subsidized bread provision during the Syrian civil war is similarly linked to pre-conflict practices. Since the early 1970s, the Syrian government supported wheat production by subsidizing most agricultural inputs and paying farmers above market value for their crops (Chemingui et al., 2010: 219). The General Establishment for Cereal Processing and Trade (HOBOOB), an agency of the Syrian Ministry of Supply and Trade, acted as the exclusive purchaser of local wheat at 140 collection centres previously operating throughout the country. The agency was also the sole flour supplier to bakeries producing subsidized bread, where retail prices were set far below production costs through a universal consumer subsidy. Not incidentally, public institutions controlled all distribution channels of the country’s ‘most important staple food’ (Chemingui et al., 2010: 189). Before the civil war, wheat provided approximately 40% of Syrian households’ caloric consumption, mostly in the form of bread. Yet equally important was bread’s symbolic role. Bashar al-Assad’s accession to the presidency in 2000 marked a decisive turn away from the previous interventionist model of development. While the proportion of government expenditure allotted to education, public employment and pensions was reduced considerably, oil and food subsidy outlays remained stable, accounting for nearly 15% of government spending prior to the outbreak of violence in 2011. Subsidized bread was one of the government’s few remaining commitments to what was once an expansive welfare system, a crucial means through which the Assad regime performed the state.

Since the onset of violence in late 2011, the Syrian government has gone to great lengths to maintain the bread subsidy in areas under its control. It has repeatedly used a $3.6 billion credit line furnished by Iran in 2013 to issue commercial tenders for wheat (Lucas, 2013). Prominent public officials pledged to use a second $1 billion Iranian credit line issued in 2015 to ‘secure the flow of essential goods and materials’ (Al-Khalidi and Westall, 2015). Despite multiple price increases triggered by inflation and a host of logistical challenges, HOBOOB exerts considerable time and effort to ensure that bakeries are open and stocked with flour. Meanwhile, various rebel groups have sought to gain civilian support by simulating elements of this long-standing welfare programme. Many of them have adjusted their military strategy and resource distribution so as to prioritize the provision of bread.^10^ Lived, everyday experiences of state administration have left deep traces in the collective memories and expectations of local populations. ‘The result is that the people are inclined to support whoever provides them services and safety’, said one Syrian aid worker (Interview 4). Certainly, the strategic decision to provide public services may be a product of ideological or tactical considerations with instrumental rationales. In addition, not every armed faction deems bread and healthcare provision to be worthy of their efforts. Nevertheless, the majority of rebel groups in Syria have tried to either directly offer or indirectly facilitate the provision of these key services to civilians. It is this process – the performative rehearsal of stateness – that Assad’s targeted bombardment seeks to destroy.

## Idlib province and city

‘*Well, some of the land might be liberated, but the sky wouldn’t let us celebrate; no, the sky was on fire*.’ (Yazbek, 2015: 5)

The province of Idlib offers an illuminating window through which to examine rebel governance. Before the outbreak of the revolution in 2011, the governorate was one of Syria’s neglected rural backwaters. Its capital, Idlib city, was the main urban outpost in what was considered a poor and conservative region with a majority Sunni population. While anti-Assad demonstrations were well attended in 2011, the province hardly seemed primed to become an epicentre of opposition activity. Peaceful demonstrations eventually turned to military means in June 2011. Low-ranking officers and soldiers in the army, disturbed by the blatant targeting of civilians, defected and joined forces with activists who had taken up arms. Various local groups – hastily organized and poorly armed – came together to comprise what would later be referred to as the Free Syrian Army. By early 2012, Syrian army units were pulling out of many of the province’s villages. Key networks of government patronage and security on which the Assad regime had relied – crony capitalists, local agents and private citizens who had benefited from their connections to the central government – fled along with the army (Lund, 2016). The sudden disappearance of these actors and institutions created a vacuum in which alternative power centres could emerge. Yet no single actor capable of unifying opposition currents stepped into the void. In particular, a lack of funding meant that officers could not afford to feed their troops or supply them with bullets, let alone provide for civilians. As Yassin-Kassab and Al-Shami note, ‘he who could provide food, weapons, and ammunition to his men was able to keep their loyalty’ (2016: 8). Better financed and armed than other opposition brigades, Islamist groups such as Jabhat al-Nusra and Ahrar al-Sham would eventually exploit the financial and institutional vacuum.^11^

In Idlib, the provision of most welfare services lies not with armed actors, but with an array of civilian-run city and town councils, which tend to draw their members from local communities.^12^ Typically, these bodies consist of a central administrative council linked to specialized offices focused on emergency relief and more typical municipal services, such as waste removal and water supply.^13^ There were as many as 144 local councils in Idlib at the beginning of 2017, including 30 larger councils that work at a city level (Heller, 2016). Some of these councils have roots in activist collectives known as Local Coordination Committees (LCCs or *tansiqiyat*), which were established by civilians after the first protests to organize media coverage and local demonstrations. Foreign donors aligned against the Assad regime soon pushed these activist networks to expand and standardize their activities. In response to such pressures, many networks formalized as local councils (*majalis mahalliye*), which ostensibly operate under the umbrella authority of the Idlib Provincial Council (*majlis muhafathat Idlib*). This provincial council is tied to the Ministry of Local Administration of the Turkey-based Syrian National Coalition, which coordinates with foreign countries and organizations to supply aid.^14^ Local councils in Idlib have been greatly strengthened by the province’s adjacency to Turkey, whose government has allowed humanitarian and development organizations to deliver supplies and assistance to the resource-scarce province. Principal among the services they offer are subsidized bread and healthcare. Persistent aerial bombing and artillery shelling by the Assad regime – coupled with mistrust amongst opposition actors and a scarcity of resources – have prevented greater coordination among councils. As a result, their collective influence remains weak: ‘Each region now has its own administration, and every village looks after itself. Everything has been turned upside down, as if every little community has become a state itself’ (Yazbek, 2015: 71).

Despite the early flourishing of opposition activity in the province, Idlib did not capture the revolution’s imagination like other parts of rebel-controlled Syria such as Homs and Aleppo. In the spring of 2015, that all changed. After months of warfare, the two most powerful rebel factions in the province – Jabhat al-Nusra and Ahrar al-Sham – combined forces. They teamed up with a collection of smaller Islamist and Free Syrian Army-affiliated groups to create an alliance called Jaish al-Fateh. Its primary goal was to capture the regime-controlled provincial capital. A carefully planned and coordinated attack on Idlib city, buoyed by an influx of foreign support spearheaded by Gulf allies, resulted in the rebels’ capture of the city in late March 2015. Not only did military success galvanize morale at a moment of stalemate across the country, effective local governance offered the prospect of a viable ‘state’ ruled by opposition forces on Syrian soil.

Only the second provincial capital to fall under opposition rule,^15^ Idlib has offered a unique challenge to the rebels in terms of governance. The tenuous alliance of Jaish al-Fateh has slowly given way to increased competition amongst the different armed and civilian groups operating in the province. Friction amongst these actors has intermittently boiled to the surface, frequently in the arena of service-provision. For example, after rebels won control of the province in 2015, Ahrar al-Sham and Jabhat al-Nusra both established separate offices to provide emergency relief and municipal services (Ahrar al-Sham, 2015). At the time, the Ahrar al-Sham-affiliated organization supported local councils by providing technocratic expertise and much-needed supplies (Heller, 2016). By contrast, the Jabhat al-Nusra-affiliated office operated independently of the local councils in areas where the latter were less established (Heller, 2016). When Jabhat al-Nusra seized the upper hand in the province in 2017, it attempted to impose its office’s standards upon the local councils. Rehearsals of statehood in Idlib are thus muddled and complex; they demonstrate aspirations to coherence rather than stabilized enactments producing state effects, as no one actor can fully implement its vision of social organization ‘on the ground’.

The significance of Idlib as a site of state rehearsal has not been lost on the Assad regime. Immediately following Jaish al-Fateh’s successful capture of Idlib in March 2015, Syrian government forces launched an intense aerial bombing campaign against the city (Syria Direct, 2015). Russian intervention in late September 2015 only escalated the frequency of such attacks. Thus far, systematic bombings have weakened the already fragile relations among the myriad opposition actors, whose alliance has always been tenuous and plagued with mistrust. ‘Every time we try to improve civil society, we run into aerial bombing that destroys these institutions’ infrastructure’, complained Idlibi judge Zaid al-Basha (Heller, 2016). ‘Planning is very difficult, sometimes impossible because of the bombs’, remarked a displaced Idlibi who had worked for his village’s local council. ‘We are forced to focus on survival instead of building our society’ (Interview 2). By targeting the built environment and material infrastructures that feed residents and care for the sick and wounded, the Assad regime has stifled two everyday practices through which local councils and rebel groups attempt to ‘perform the state’.

## Our daily bread

‘*Everyone eats bread in Syria*.’ Tarif al-Sayyed Issa (Lund, 2015)

In Idlib, rebel groups engage in performative practices that seek to win over followers to their preferred vision of social and political organization. They have done so by borrowing and mimicking technologies of governance from the incumbent authority they seek to replace. The provision of bread is one of the most important practices in this pursuit. It should come as little surprise that the first measure Jaish al-Fateh took after securing control of Idlib city in March 2015 was to reopen its bakeries (Jama’a, 2015; Abdulrahim, 2015). Military agreements do not always extend beyond the battlefield, however, as Jabhat al-Nusra sought to independently control the distribution of flour in Idlib city before ceding control to other rebel groups in the alliance (Lund, 2015). In its attempts, Jabhat al-Nusra followed the example of Ahrar al-Sham, which already ran a number of its own bakeries in other provincial towns (Yazbek, 2015: 54). Both groups’ efforts to control resources while fostering alliances suggest their vacillating relationship with local councils, which they alternatingly support and undermine (Al-Tamimi, 2017).

Despite the latent – and sometimes outright – animosity, rebel groups in Idlib have often coordinated with local councils to ensure the provision of bread, granting them differing levels of autonomy.^16^ In many cases, rebel groups have found it beneficial for their reputations to allow local councils to continue functioning, albeit under the insurgents’ supervision (Favier, 2016). By adjusting their military strategy to ensure that the necessary supply routes remain open and deliveries made, armed groups both benefit financially via the taxation of goods and secure their reputations as guarantors of stability. The local councils have little interest in adopting a confrontational approach towards rebels, whose brigades provide security and a semblance of order – even if the latter may occasionally use control over bread as a way of undercutting the former’s claim to authority (Munif, 2017). One aid worker described these dynamics succinctly: ‘Part of what makes a local council successful is that it can navigate relationships with armed groups, not that it insulates itself from them’ (Heller, 2016). Ahrar al-Sham is often perceived to be more amenable to such arrangements. Local councils can also exert leverage through their access to vital international aid, which Islamist groups such as Jabhat al-Nusra cannot officially obtain because they are designated ‘terrorist’ organizations by key foreign donors. In the realm of welfare provision, rebel groups and local councils have thus largely found their relationship to be mutually beneficial, if rife with suspicion. Nevertheless, continuous regime bombing of bakeries, wheat fields and flourmills has disrupted the stability that opposition actors have tried to foster via bread provision.

Already by April 2013, a Human Rights Watch report (2014) found that regime forces were deliberately targeting bakeries and bread lines in rebel-held areas with both artillery and airstrikes. Since the loss of the provincial capital in 2015, regime attacks on bakeries in Idlib have only increased. In July 2015, Syrian government warplanes struck a bakery in the middle of Idlib city, killing at least five civilians (Syria News Desk, 2015). A year later, pro-regime warplanes destroyed the last major bakery in the city. In November 2016, Syrian government planes bombed the largest bread oven in southeast Idlib province (Jida’an and Abdul Qader, 2016). ‘It was the only automated bread oven in the area and supplied around 74 towns and villages, all of which are under opposition control’, explained Jaber Abu Mohamed, the spokesperson for the local council in the area (Bakur, 2016). Earlier in 2016, the Syrian government launched a series of aerial attacks on the bakeries in and around the town of Binnish, which is home to one of the most effective local councils in Idlib province. Binnish, not coincidently, has been the recipient of humanitarian assistance from multiple international organizations in the form of wheat and bread (Karam Foundation, 2016). Targeted bombardment of Binnish’s service infrastructure is indicative of a larger trend. The rebel-held areas most prone to regime attacks are frequently those that receive the most humanitarian aid, and, in turn, are the most active in ‘performing the state’.

A Russian bombing of an Idlibi bakery and flourmill in November 2015 illustrates the numerous actors that shape bread provision in the province. The bakery was established in the town of Saqareb by one of Turkey’s largest humanitarian organizations, The Foundation for Human Rights and Freedoms and Humanitarian Relief (IHH). The organization, which is closely tied to the avowedly anti-Assad Turkish government, has played a major role in supporting rebel governance during the conflict by providing flour and other subsistence goods to local councils. Prior to its demolition, Saqareb’s main bakery produced around 16,000 loaves of bread a day, serving approximately 50,000 people (ThinkPol, 2015). Interestingly, the warplanes targeting the bakery made a concerted effort to spare civilian lives. ‘Russian jets first fired at positions close to the bakery as a warning’, said IHH officials in a statement following the attack, ‘but after people left the bakery, they demolished the building’ (ThinkPol, 2015). It was not the activists or employees running the bakery who were most threatening but rather the capacity of local groups to enact state performances that proved most menacing to the Assad regime.

The bombing of Saqareb also illuminates how rebel attempts to perform the state are intimately linked to and heavily dependent on external actors.^17^ Throughout Idlib province, the routinization of bread provision hinges on access to foreign aid. Local councils and rebel groups have neither the funds nor the administrative apparatus to engage in such efforts alone. When international donors and foreign allies halt the flow of flour and other resources into the province, Syrian rebels cannot make up the deficit; their attempts to ‘rehearse the state’ are thus tenuous and highly dependent on outside assistance (Martínez and Eng, 2016). Of course, the opposition is not alone in its reliance on external actors. The Assad regime’s campaign to destabilize rebel governance has been greatly aided by Russian support, especially in its aerial bombardment campaign. Performing the state in Syria is far from a strictly local concern: it has become a transnational affair.

## Hospitals and healthcare provision

‘*As soon as you cross the border [into Syria], you are vulnerable to aerial bombing by the Syrian Air Force*.’

Doctors Without Borders (as quoted in Syria Deeply, 2013)

If the Syrian government’s bombing of bakeries represents a challenge to one fundamental premise on which opposition groups rest their claim to authority – the capacity to provide a minimal level of subsistence – the deliberate bombardment of hospitals represents another direct challenge to a key rebel claim: the ability to ensure physical security and health. The overt politicization of healthcare facilities is a tactic that the Assad regime has pursued since the outbreak of the conflict. During protests in 2011, pro-opposition activists often refused to seek treatment in national hospitals out of fear that Syrian intelligence would target them there (Taub, 2016). In mid-2012, a counter-terrorism law was passed in parliament that effectively criminalized medical assistance to members of the opposition. As the conflict escalated, so did the deliberate targeting of medical infrastructure and personnel. During the first two years of the civil war (2011–13), the withdrawal of government forces and employees from opposition-held cities and towns resulted in the rebels’ appropriation of existing hospitals to treat the wounded. Yet the Assad regime retained the physical coordinates of these facilities, previously registered by the Syrian Ministry of Health (Physicians for Human Rights, 2016a). Many of these public hospitals were targeted and hastily destroyed. In August 2013, a UN commission found that the Assad regime had deliberately assaulted hospitals and medical units to deprive anti-government militias and their perceived supporters of access to medical assistance. A pattern of bombings in the opposition-held areas of Homs, Aleppo and Dar’a led the commission to conclude that ‘the denial of medical care as a weapon of war is a distinct and chilling reality of the war in Syria’ (United Nations Human Rights Office of the High Commissioner, 2013). As of October 2016, the Syrian government and its allies had conducted approximately 90% of total attacks against medical facilities, notwithstanding international condemnation (Physicians for Human Rights, 2016b). ‘The regime is absolutely deliberate in their bombing’, said a communications director at one Idlib hospital (Schuster et al., 2016).

The increasing frequency of strikes since 2015 against medical facilities in Idlib follows a similar pattern to that of bakery bombings – increased targeting of areas where rebel governance is most successful. Doctors Without Borders (2016) recorded a significant spike in attacks on medical facilities in Idlib and other parts of northern Syria in May and June 2015 following major opposition victories in the area, including the capture of Idlib city. Similarly, Physicians for Human Rights documented 122 attacks on hospitals in 2015 alone – more than a third of the total since the beginning of the war (Brown, 2016). In 2016, regime and Russian warplanes attacked seven of the ten major hospitals in the province, according to a member of the Idlib Provincial Council (Schuster et al., 2016). The Idlib city hospital mentioned in the Introduction was among those targets. The severity of that attack, which came as part of a larger series of overnight strikes on the province in May 2016, led to the precautionary closure of all other service-providing centres in the area (Mahmoud, 2016). Just two months later, Syrian and Russian planes bombed medical facilities in the provincial towns of Millis and Sarmen. Both facilities were clearly marked as medical and no combatants operated in the area.

These onslaughts hinder insurgents’ military capabilities, but that is not their sole or most important effect. By making life difficult for those under opposition rule, the Assad regime’s bombing campaign worsens the damage of physical wounds and undermines morale amongst civilians, as the wounded and sick are denied treatment. Most importantly, it disrupts rebel attempts to perform the state, preventing the routine practices that seek to normalize their rule. Syrian writer Samar Yazbek reflects upon this in her record of time spent in opposition-held towns in Idlib province: ‘The people here lived side by side with death. This was no metaphor, but reality. They didn’t think about any big issues, they weren’t interested in understanding the military situation or the political context; they had no space to think. All they could do was struggle to survive’ (Yazbek, 2015: 131). For those Syrians in need of recurring or emergency treatment, the options are increasingly limited: move to regime-held areas or flee across borders as refugees. One resident of Maarat al-Numan, having recently fled to Lebanon, highlighted the dilemmas caused by the rebels’ struggles to provide healthcare: ‘All of my family supported the revolution but we had to leave. Two of us have Type 1 diabetes, some of the children have developed typhoid and hepatitis and need urgent care. We had no choice, healthcare facilities were all destroyed. So many like us have left’ (Interview 1). The persistence of regime bombardment stifles rehearsals of stateness even among civilians with anti-Assad sympathies.

Rebel groups and their foreign backers have tried to adjust to the persistent aerial assaults. This is not only because of their importance to the livelihoods and security of ordinary civilians, but also because they comprise a critical arena in which insurgent groups prove themselves against each other and the Assad regime. While many of the major hospitals in Idlib are no longer operating, out of fear of new attacks, international medical organizations like the Syrian American Medical Society (SAMS) and Doctors Without Borders have supported smaller, clandestinely located clinics. One Idlib-based maternity hospital opened in 2016 is based in an abandoned office building tucked away in a residential area so as to avoid drawing attention to itself. ‘This hospital is a huge accomplishment for the medical community in our town’, said a local resident. ‘Of course, there is the fear that we could be bombed any moment’ (Schuster et al., 2016). Other medical organizations working with rebel groups have tried to adjust to regime bombings by building medical facilities underground. ‘It certainly doesn’t guarantee complete protection for our doctors and nurses, but, quite frankly, it’s the best option we got’, said Dr Manthour Khaleel, a spokesperson for the Idlib Health Directorate, the administrative branch responsible for medical activity in the province (Nassar et al., 2016). Even underground facilities were not completely safe, as the Assad regime began to deploy bunker-busting missiles. ‘When I am in the hospital, I feel like I am sitting on a bomb’, explained Dr Tennari, the director of SAMS sponsored hospital in Idlib. ‘It is only a matter of time until it explodes’ (SAMS, 2016). Faced with an unceasing barrage, insurgents, local councils and their international partners constantly adapt in order to continue offering healthcare services. Consequently, they have not been able to invest in permanent medical facilities and other infrastructures that could normalize healthcare provision.

These efforts are not unique to Idlib. Nearly all opposition cities and towns with a modicum of successful administration have been blitzed, including the East Ghouta suburbs near Damascus, East Aleppo and Dar’a province, as well as the Islamic State’s former stronghold in Raqqa (Daher, 2016). This indicates the extent to which rebel governance has been and continues to be perceived as a major hazard to the Assad regime’s authority. This is perhaps best exemplified by the three cantons under YPG rule. These have flourished in the absence of aerial bombardment, as Kurdish rule is perceived by the Assad regime as a less dire threat than the mainstream opposition’s claims to authority, a status quo that the YPG strives to maintain. As the military dynamics in the country shift, however, the regime’s perceptions of that threat may change. In contrast, nominally opposition-controlled territories with effective local councils and rebel groups such as Idlib province have been the most affected by the regime’s tactics. Many have had to scale back public services in territories that have slowly been emptied of their population. The sometimes coordinated, sometimes antagonistic roles that local councils, armed brigades, Islamist groups and international aid organizations all play in the provision of bread and healthcare in this province speak to the shared recognition of these services’ significance. Yet regime tactics have prevented the routinization and expansion of these efforts, further exacerbating differences between the groups and impeding the effectiveness of ‘state performances’ as a strategy of resistance. While the actors and internal dynamics of rebel competition in Idlib may be idiosyncratic to the province, pre-conflict welfare practices and the expectations they engender amongst civilians are not: performing the state via the routinization of bread provision and healthcare has been a crucial fulcrum of Syria’s civil war.

## Conclusion

Similar to other non-state actors lacking but aspiring to de jure sovereignty, rebel groups in Syria have provided welfare so as to bolster local support and legitimize their rule (Wilson and McConnell, 2015). Their efforts to ‘perform the state’ have been composed of a series of carefully calculated and deliberate actions meant to resist, or at least problematize, the Assad regime’s sovereignty. We believe these everyday practices are one of the Assad regime’s main concerns and help explain the patterns of aerial bombardment seen since the conflict’s outset. Certainly, this policy has other tactical objectives: pushing local civilians to pressure the fighters in their midst towards surrender, signalling to Syrians elsewhere the price of continued resistance and displacing pro-opposition populations that might pose challenges to the Assad regime in the future. Calculated airstrikes also maximize the value of limited manpower and military supplies. The Syrian government’s campaign, however, is not merely about committing wanton violence or achieving military gains. In the monogamous spirit of modern states, the Assad regime deems it crucial to eradicate rival performances of statehood. And in that it has been successful. We do not wish to claim that the Assad regime’s attacks have been the sole reason for the opposition’s failure to construct a coherent polity. In Idlib, rebel governance has been fragile and limited for many reasons. The absence of a coordinated donor strategy, competition amongst implementing agencies and rebel groups and pervasive mistrust have helped prevent the construction of sustainable modes of rule. But we do contend that the regime’s attacks have significantly contributed to this outcome, thus achieving their main goal insofar as no unified state-like actor or governance structure has successfully emerged from the debris.

Undoubtedly, many aspects of the Syrian conflict do not lend themselves to any sort of reasonable generalization. Yet we believe that our approach helps highlight dynamics crucial to the study of civil war. First, by offering grounded examples of how the state, as an ‘effect’, is (re)produced, we have offered a framework through which to examine rebel governance beyond its strictly quantifiable characteristics. Second, we have foregrounded the provision of welfare so as to demonstrate that it is through the calling into existence of a historically conditioned set of obligations and expectations between service providers and their intended recipients that opposition groups in Idlib seek to position themselves as state-like. Rebel governance hinges on everyday measures that gain their force from these locally grounded histories. Finally, a performative approach brings to the fore the political impact of the Assad regime’s unswerving *modus operandi*: destroying the built environment and material infrastructures crucial to welfare provision. It makes clear what is at stake in such targeted demolition: preventing the rebels’ aspirational rehearsals of stateness from becoming fully fledged state performances. Syrians across the country have made these insights painfully obvious; it is time that the civil war scholarship incorporated their insights.

## Notes

1. There remains speculation over who conducted the aerial raid. Although Russian government spokespersons have denied involvement, the Turkish government has attributed it with responsibility for the raid.

2. We use the terms ‘rebels’ and ‘insurgents’ interchangeably throughout this article. We define an insurgency as a prolonged violent struggle through which organized groups seek to obtain political objectives (independence, autonomy, takeover of public institutions) against the incumbent political authority.

3. It also exposes how vulnerable provisional alternatives are in the face of aerial bombardment.

4. This is not always the case. During predatory rebellions, such as the one headed by Charles Taylor in Liberia, insecurity and arbitrary rule, rather than routinization facilitate governance. For more, see Reno (2015).

5. Statehood is characterized by historically and locally specific practices that become naturalized or assumed in relation to sovereign authority over a given territory. These practices are shaped by dynamics and arenas as diverse as international law, domestic patterns that work to produce political authority and legacies of rule, among many others.

6. The Islamic State is not exempt from this assertion (see Hamdan, 2016).

7. Also mentioned, although less frequently, were electricity, residential water and sanitation.

8. For example, life expectancy at birth increased from 56 years in 1970 to 73.1 years in 2009; infant mortality dropped from 132 per 1,000 live births in 1970 to 17.9 per 1,000 in 2009; under-five mortality dropped significantly from 164 to 21.4 per 1,000 live births (Kherallah et al., 2012).

9. Government expenditure on health decreased from 4.9% of GDP to 3.4% between 2000 and 2010 (Taleb et al., 2015).

10. Similar efforts were made in the semi-autonomous Kurdish cantons. For more, see Knap et al. (2016: 93).

11. Jabhat al-Nusra changed its name to Jabhat Fateh al-Sham in 2016 in an attempt to separate itself from its Al-Qaeda affiliations. It later changed its name again in 2017 to Haya’t Tahrir al-Sham in a merger with smaller armed Islamist groups. These variations reflect a fluid political scene in which groups and their affiliations frequently shift according to changing military circumstances. In order to avoid confusion, this article will continue to refer to the group as Jabhat al-Nusra.

12. In some cases they were established through democratic means, while in others they reflected informal family and tribal quotas.

13. This process developed differently in the three cantons (Afrin, Jazira and Kobani) intermittently under control of the People’s Protection Units (YPG). For more on local institutions in the Kurdish cantons, see Knap et al. (2016: 84–121).

14. The National Coalition has gone to great lengths to integrate local councils into its administrative structures. Its efforts are viewed by many as a way of overcoming the organization’s lack of legitimacy ‘on the ground’. Groups established in Turkey dedicated to assisting the local councils include the Assistance Coordinating Unity (December 2012), the Local Administration Council Unit (March 2013) and finally, the Ministry of Local Administration, Refugees and Humanitarian Relief created by the General Directorate for Local Councils (March 2014). Donor rivalries and internal conflicts have delayed efforts to consolidate local councils under a uniform organizational structure.

15. The other being Raqqa, the former de facto Syrian capital of the Islamic State.

16. Their relationships are frequently built on previously existing networks ranging from familiar and professional affiliations to solidarity between neighbours, tribes and kin.

17. For a personal account of bombings in Saraqeb, see Yazbek (2015: 58–72).

## Interviews cited

1. Syrian refugee, 12 July 2015, Beirut, Lebanon.
2. Syrian refugee, 14 July 2015, Beirut, Lebanon.
3. Syrian refugee, 18 July 2015, Beirut, Lebanon.
4. Syrian aid worker, 28 March 2016, Beirut, Lebanon.
